# Reexamination of the Sida Micrantha Mosaic Virus and Sida Mottle Virus Complexes: Classification Status, Diversity, Cognate DNA–B Components, and Host Spectrum

**DOI:** 10.3390/v16111796

**Published:** 2024-11-19

**Authors:** Marcos Silva de Queiroz-Ferreira, Luciane de Nazaré Almeida dos Reis, Maria Esther de Noronha Fonseca, Felipe Fochat Silva Melo, Ailton Reis, Leonardo Silva Boiteux, Rita de Cássia Pereira-Carvalho

**Affiliations:** 1 Department of Plant Pathology, University of Brasília (UnB), Brasília 70910-900, DF, Brazil; marcsqferreira@gmail.com (M.S.d.Q.-F.); lucianealmeidareis@outlook.com (L.d.N.A.d.R.); ffochatsm@hotmail.com (F.F.S.M.); leonardo.boiteux@embrapa.br (L.S.B.); 2 Embrapa Vegetable Crops (Hortaliças), National Center for Vegetable Crops Research (CNPH), Brasília 70351-970, DF, Brazil; maria.boiteux@embrapa.br (M.E.d.N.F.); ailton.reis@embrapa.br (A.R.)

**Keywords:** breeding, *Solanum lycopersicum*, high-throughput sequencing, single-stranded DNA viruses, Malvaceae, *Geminiviridae*

## Abstract

Sida mottle virus (SiMoV) and Sida micrantha mosaic virus (SiMMV) are major Brazilian begomoviruses (*Geminiviridae*). However, the range of DNA–A identity of isolates of these viruses (81–100%) is not in agreement with the current criteria for *Begomovirus* species demarcation (<91%). To clarify this putative classification problem, we performed a comprehensive set of molecular analyses with all 53 publicly available isolates (with complete DNA–A genomes) designated as either SiMoV or SiMMV (including novel isolates obtained herein from nationwide metagenomics-based studies). Two well-defined phylogenetic clusters were identified. The SiMMV complex (*n* = 47) comprises a wide range of strains (with a continuum variation of 88.8–100% identity) infecting members of five botanical families (Malvaceae, Solanaceae, Fabaceae, Oxalidaceae, and Passifloraceae). The SiMoV group now comprises eight isolates (90–100% identity) restricted to Malvaceae hosts, including one former reference SiMMV isolate (gb|NC_077711) and SP77 (gb|FN557522; erroneously named as “true SiMMV”). Iteron analyses of metagenomics-derived information allowed for the discovery of the missing DNA–B cognate of SiMoV (93.5% intergenic region identity), confirming its bipartite nature. Henceforth, the correct identification of SiMoV and SiMMV isolates will be a crucial element for effective classical and biotech resistance breeding of the viral host species.

## 1. Introduction

*Begomovirus* is the largest genus in the *Geminiviridae* family, thus far encompassing ≈ 445 species [1]. These single-stranded DNA (ssDNA) viruses have either monopartite or bipartite circular genomes. The bipartite species have two genomic components (DNA–A and DNA–B with ≈ 2600 nucleotides each) and are predominant in the Neotropical areas. The DNA–A and DNA–B genomic components share a common (intergenic) region (CR) of ≈200 nucleotides that displays high identities (greater than 90%) across cognate components of the same viral species [1,2]. Begomoviruses can have altogether six to ten open reading frames (ORFs). The ORFs in the DNA–A component (in the viral-sense) are the AV1, which encodes the coat protein, and the AV2 gene (present in Old World begomoviruses) that encodes a movement protein [2]. In the complementary-sense, AC1 codes for the replication-associated protein [1,2], AC2 encodes the transcriptional activator protein TrAP [3], AC3 encodes the replication enhancer protein REn [2,4], and the gene product of AC4 is associated with symptom development, viral movement, and replication [5,6,7]. Novel ORFs (and their functions) have been more recently characterized, including AC5 described in mungbean yellow mosaic India virus as a determinant of pathogenicity and suppressor of gene silencing [8,9]; the mitochondria-associated AC6/C6 gene product was detected in isolates of tomato leaf curl China virus [10], whereas the AC7/C7 gene was described in tomato yellow leaf curl virus (TYLCV) as a suppressor of gene silencing [11]. The highly conserved, Golgi-associated AV3 gene product is essential for TYLCV infection, acting as a post-transcriptional RNA silencing suppressor, facilitating virus replication and systemic spread [12].

The taxonomic criteria of the genus *Begomovirus* at the species level have been constantly reexamined over the years, being currently based upon analyses combining MUSCLE alignments and the Sequence Demarcation Tool (SDT) [13]. A new set of criteria for taxon differentiation was established, defining a novel *Begomovirus* species as the one displaying the complete sequence of the DNA–A component with identity below 91% with all previously known viruses within this genus [13].

Sida mottle virus (SiMoV = *Begomovirus sidavariati*) and Sida micrantha mosaic virus (SiMMV = *Begomovirus sidamicranthae*) are the major Malvaceae-associated begomoviruses from Brazil. The DNA–A component of the first SiMoV isolate was deposited in GenBank in the year 2002 (gb|AY090555 = NC_004637), but without information about the original host species, as well as its cognate DNA–B component. After that (2003), two partial sequences (ranging from 677 to 693 nucleotides) of SiMoV isolates from soybeans were deposited (AY436328 and AY444554). In 2012, two new SiMoV isolates infecting *Sida santaremensis* were characterized and their complete DNA–A sequences were deposited in GenBank (JX871377 and JX871378).

The first formal report of SiMMV was carried out in 2004 with samples collected in Campinas, São Paulo–SP, Brazil [14]. The complete DNA–A sequences of two isolates were obtained: A1B3 (gb|AJ557450 = NC_077711 with 2659 nucleotides), and A2B2 (AJ557451 with 2675 nucleotides). The complete DNA–B sequences were simultaneously obtained for the isolate A1B3 (NC_077712 with 2629 nucleotides) and from the isolate B1 (AJ557452 with 2652 nucleotides). Almost simultaneously, a novel SiMMV isolate (named as MG-Bi2) was detected in association with tomato (*Solanum lycopersicum*) plants in São Joaquim de Bicas–MG, Brazil [15]. However, MG-Bi2 displayed 87% identity to SiMoV and 81% identity to SiMMV, being, in fact, the first description of a tomato yellow spot virus (ToYSV) isolate [15]. In another work published in 2010, the complete sequences of the DNA–A (2665 nucleotides; FN557522) and DNA–B (2632 nucleotides; FN557523) components were obtained for the isolate SP77, which was named the “true SiMMV” [16]. Somewhat surprisingly, our initial BLAST analyses indicated that both A1B3 (NC_077711) and SP77 (FN557522) isolates are apparently with erroneous designation in GenBank since they are more likely strains of the reference isolate of SiMoV (NC_004637), with overall identities of 91.77 and 92.03%, respectively. The complete DNA–A (AJ557451 = NC_005330) and DNA–B (AJ557453 = NC_005331) sequences of the SiMMV isolate A2B2 were latter established as additional reference sequences for SiMMV.

In this current scenario, levels of identity of the DNA–A genome across isolates reported in GenBank as SiMMV and SiMoV are ranging from 81.9 to 100%, demonstrating a taxonomical and/or nomenclatural inconsistency of these begomoviruses. In addition, our initial set of analyses indicated a close genetic relationship (91.77% identity) of the “true SiMMV” and the reference SiMoV isolate (AY090555 = NC_004637), which generated uncertainties about their taxonomic status, according to the current criteria of *Begomovirus* species classification [1]. SiMMV isolates have been predominantly reported in Malvaceous weeds, but also in cotton, as well as in a wide range of economically important hosts in the Neotropics [17,18,19,20,21,22]. On the other hand, relatively few novel reports of SiMoV have been released since 2012.

To clarify this putative classification problem, we carried out extensive analyses with all SiMMV and SiMoV accessions with complete DNA–A genomes available in GenBank. Our objective was to reveal the diversity present in these two Malvaceous-infecting viral complexes. In addition, we provided updated information about the relative prevalence, host range, and the geographical distribution of both viruses across all Brazilian regions via high-throughput sequencing (HTS) analysis of samples collected in nationwide surveys from 2001 to 2020.

## 2. Materials and Methods

### 2.1. Leaf Samples from Malvaceae Weeds with Begomovirus-like Symptoms

Seventy-nine (79) leaf samples of Malvaceae weeds showing symptoms of begomoviruses (golden and bright yellow mosaics) were collected in 11 states across all five macrogeographical regions of Brazil (Table 1). The samples were collected from 2001 to 2020 in the South (*n* = 1), North (*n* = 21), Northeast (*n* = 22), Midwest (*n* = 21), and Southeast (*n* = 14) (Table 1). These samples were selected to cover a broader geographical and chronological snapshot.

### 2.2. DNA Extraction

DNA total was extracted from each individual sample by using a modified protocol that includes CTAB buffer plus organic solvents [23]. The DNA quantification of the samples was performed using a NanoDrop^®^ spectrophotometer (Thermo Fisher Scientific, Waltham, MA, USA). The DNA integrity was verified through 1% agarose gel electrophoresis.

### 2.3. Enrichment of Circular DNAs via Rolling Circle Amplification—RCA

The total genomic DNA obtained from each sample was then used as a template for Rolling Circle Amplification—RCA [24]. The purified DNAs were also employed as templates in polymerase chain reaction (PCR) assays aiming to confirm the presence of begomoviruses in the samples. Amplicons derived from a segment of the DNA–A component (≈1200 bp) were obtained using the primer pair “PAL1v1978/PAR1c496” [25].

### 2.4. HTS and Sequence Analyses

The RCA products of all samples were grouped into a single pool (named as BP1). The library was prepared using the kit Nextera XT, which was analyzed using one Illumina^®^ NovaSeq 6000 sequencing platform (Illumina Inc., San Diego, CA, USA) at Agrega (Porto Alegre, RS, Brazil) using 150 bp paired-end reads. The reads obtained were initially assembled into contigs by using the CLC Genomic Workbench 23.0.2 program (CLC bio, Aarhus, Denmark). After assembling the contigs, the “Map To Reference” tool available in the Geneious^®^ R11.1.5. program [26] was used to map the reads. An overlap identity of 100% was employed as the criterion to confirm if a given read corresponded to either SiMMV or SiMoV. The contigs were compared with sequences deposited in the GenBank database (https://www.ncbi.nlm.nih.gov/, accessed on 25 October 2024) using the BLASTn algorithm. MUSCLE alignments were performed using the Geneious^®^ 11.1.5 program. Annotation of ORFs was based upon the reference genomes.

### 2.5. Phylogenetic Analyses

In view of the potential inaccuracy regarding the taxonomic status of these group of begomoviruses, we carried out a phylogenetic analysis of all 53 isolates with complete DNA–A sequences (data recovered in May 2024 in GenBank) classified as either SiMMV or SiMoV (Supplementary Table S1). This analysis was performed with the MUSCLE alignment and the software Sequence Demarcation Tool—SDT [27]. Phylogenetic reconstructions were performed using the IQtree program using the Bayesian method, model TIM3 + F + R3 with 1000 bootstrap replications, as previously described [28]. Evaluations of iterons and conserved motifs in the CR among SiMMV and SiMoV isolates were performed as previously described [29].

### 2.6. PCR Conditions Used to Detect SiMMV and SiMoV Isolates in Individual Samples

SiMMV-specific [30] and SiMoV-specific (present work) PCR primer pairs were designed (Supplementary Table S2). These primer pairs contain polymorphisms at the 3′ terminus that ensure their specificity. They were used to confirm the presence of these begomoviruses in each individual DNA sample. Reactions were carried out in a total volume of 12.5 μL, containing the following components: *Taq* polymerase buffer [10×], 1.25 μL; MgCl_2_ [50 mM], 0,4 μL; dNTPs [10 mM], 0.25 μL; forward and reverse primers [10 mM], 0.25 μL; Milli-Q water, 8.0 μL; and *Taq* polymerase [5 U/μL], 0.10 μL. The 34 amplification cycles were divided into the following steps: initial denaturation (94 °C for 3 min), denaturation (94 °C for 30 s), annealing for 45 s (at 58 °C for SiMMV and at 55 °C for SiMoV), extension (72 °C for 3 min), and final extension (72 °C for 10 min). The amplicons generated were visualized in an agarose gel (1%) stained with ethidium bromide and under UV light in a trans-illuminator and photodocumented. The amplicons of all isolates were purified and partially Sanger-sequenced.

### 2.7. Analysis of Common (Intergenic) Region Motifs and Conserved Regions, and Search for the ORFs V3, AC5, AC6, and AC7 in the SiMMV and SiMoV Genomes

During the analyses of the HTS sequences from the pool of 79 Malvaceae samples, three novel isolates of SiMoV were detected via genome assembly. An analysis of putative DNA–A and DNA–B cognates of SiMoV was performed by aligning the common region of both components according to previous work [29,31]. TATA-BOX, nonanucleotide, iteron searches, and iteron-related domain (IRD) analyses were performed. Differences between SiMMV and SiMoV species were analyzed for the structural helix 4 motif, which is conserved across geminiviruses [32]. The near-palindromic [ACTT–(N7)–AAGT] sequence was also analyzed in the DNA–A component. This structural element (present in the promoter region of coat protein-coding gene) is conserved among *Geminiviridae* members [33]. The presence of the novel ORFs AV3, AC5, AC6, and AC7 [7,8,9,10,11,12] was also analyzed with the Geneious^®^ 11.1.5 program.

### 2.8. Analysis of Recombination Events via RDP5

The sequences of the SiMMV and SiMoV isolates were aligned using pairwise MUSCLE multiple alignment with the help of the software RDP5 program aiming to detect putative recombination events. Recombination events were considered reliable only if they were detected by at least four out of the seven methods implemented by the program [34].

## 3. Results

A total of 16,079,516 paired-end reads (150 bp) was generated from a pool of foliar samples of Malvaceae weeds, encompassing 10.679 contigs, with 207 of them corresponding to genomic segments of viruses as indicated through BLASTn analysis. Eight contigs were related to SiMMV with overall identities over 97%. Therefore, only one of them was included in the phylogenetic analysis. Three sequence variants of SiMoV were detect via HTS in a mixture of foliar samples (named herein as ES–076 isolate), which were collected during surveys in Espírito Santo State, Brazil. The remaining symptomatic samples were infected by different begomoviruses.

**Figure 1:**
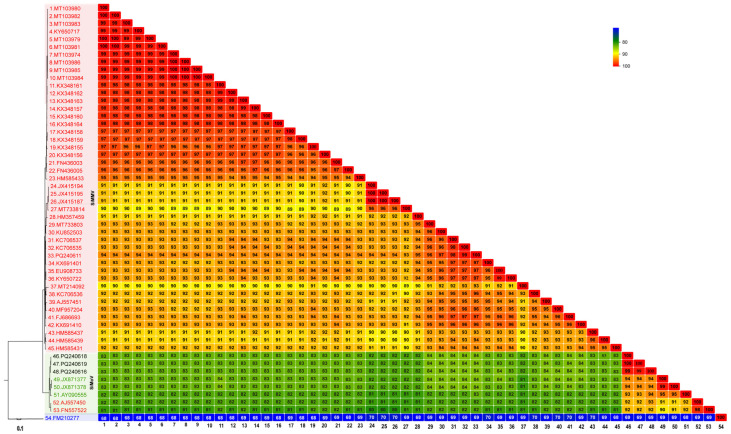
Phylogenetic tree and Sequence Demarcation Tool (SDT) analysis of DNA–A components showing the phylogenetic distances among Sida mottle virus (SiMoV = *Begomovirus sidavariati*) and Sida micrantha mosaic virus (SiMMV = *Begomovirus sidamicranthae*) isolates. These isolates are identified by their accession numbers in GenBank. The accession numbers of isolates classified/named as SiMMV in GenBank are the following: JX415195, JX415194, JX41518, MT733814, HM357459, MT733803, KU852503, KC706537, KC706535, KX691401, PQ240611, KY650722, EU908733, FN557522 (=SP77 isolate), AJ557450 (NC_077711 = A1B3 isolate), FJ686693, MT214092, KC706536, AJ557451 (=A2B2 isolate), MF957204, KX691410, MT103982, MT103980, MT103983, KY650717, MT103979, MT103981, MT103986, MT103974, MT103985, MT103984, KX348162, KX348161, KX348163, KX348157, KX348160, KX348164, KX348159, KX348158, KX348155, KX348156, FN436005, FN436003, HM585433, HM585431, HM585439, and HM585437. Two isolates: FN557522 (=SP77 isolate) and AJ557450 (NC_077711 = A1B3 isolate) were reclassified as SiMoV in the present work. The isolates classified/named as SiMoV are the following: PQ240619, PQ240618, PQ240616, AY090555 (=NC_004637), JX871378, and JX871377. Tomato leaf curl virus (FM210277) was used as the outgroup. GenBank accessions PQ240619, PQ240618, and PQ240616 were characterized in the present work.

PCR assays with a set of previously selected virus-specific primers were performed to validate the HTS-derived data. Of the 79 samples, 41 samples were positive for SiMMV. (Supplementary Table S2). These results were confirmed via partial sequencing of the corresponding amplicons. The results are illustrated in the Supplementary Table S3. Two well-defined phylogenetic clusters were identified (Figure 1). The SiMMV complex (*n* = 47) comprises a wide range of strains (with a continuum variation of 88.8–100% identity) infecting members of five botanical families (Malvaceae, Solanaceae, Fabaceae, Oxalidaceae, and Passifloraceae). The SiMoV group now comprises eight isolates (90–100% identity) restricted to Malvaceae hosts, including one former reference SiMMV isolate (gb|NC_077711) and SP77 (gb|FN557522; erroneously named as “true SiMMV”) (Figure 1; Supplementary Table S1).

Evaluations of iterons and iteron-related domains (IRDs) discriminated the isolates into five subgroups displaying three distinct iterons (GGGGT, GGGTA and GGTAG). A subset of isolates identified as SiMMV subgroup #1 shared identities ranging from 93 to 99%. Subgroup #1.1 comprised 18 SiMMV isolates with iteron GGGGT, which displayed the same IRD (MPPPKRFKIS). The following isolates are part of this group: FN436003 (described as infecting *Sida rhombifolia* in Mato Grosso do Sul–MS, Brazil) [35]; FN436005 (in *S. micrantha* in MS, Brazil) [35]; KX348155, KX348157, KX348160 KX348161, KX348162, KX348163, and KX348164 (in *Sida* species in Brazil) [36]; and MT103974, MT103979, MT103980, MT103981, MT103982, MT103983, MT103984, MT103985, and MT103986 (described as infecting *Passiflora edulis* in Bahia–BA, Brazil). On the other hand, the subgroup #1.2 comprised four isolates of SiMMV with iteron GGGTA with IRD (MPPPKRFKIS) viz. HM585433 in *Sidastrum micranthum* in Bolivia [37] and KX348156, KX348158, and KX348159 in *Sida* species in Brazil [36].

Subgroup #2 shared identities from 90 to 94%. Subgroup #2.1 included 18 isolates described as SiMMV with iteron GGGGT and IRD (MPPPKRFKIS) viz. AJ557451 reported in *S. micrantha* in Brazil [14], FJ686693 in soybeans in Brazil [17], HM585431, HM585437, and HM585439 in *S. rhombifolia* in Bolivia [37], KC706535, KC706536, and KC706537 in tomatoes in Brazil [38], KU852503 in soybean in Central Brazil, KX691401 in *S. spinosa* in Northeast Brazil, KX691410 (in *Sida* sp. in Northeast Brazil), KY650717, and KY650722 (in *Oxalis* species in Brazil [20], MT214092 in tomato in Brazil [31], and MT733803 in tomato in Brazil [39]. The three novel isolates described herein (named as PQ240611, PQ240612, and PQ240614) were collected in the Espírito Santo–ES state (Southeast Brazil) and allocated to this group. Subgroup #2.2 comprised four isolates described as SiMMV with iteron GGTAG and IRD (MPSAPKRFQI) viz. JX415187 infecting *Sida* sp. in Central Brazil [40], JX415194 and JX415195 in *S. santaremensis* in Central Brazil [40], and MT733814 in tomato in Brasília-DF [39].

Subgroup #3 comprised the SiMoV-related isolates, including the original isolate erroneously described as SiMMV (FN557522 = SP77) infecting *S. micrantha* in Campinas, São Paulo–SP, Brazil [16]. Three isolates originally described as SiMoV viz. AY090555 (host not available and collected in Viçosa, Minas Gerais, MG, Brazil), JX871377, and JX871378 (both in *S. santaremensis* also in Viçosa–MG, Brazil) were also allocated to this group together with three sequence variants, PQ240619, PQ240618, and PQ240616 (all three derived from the present work). It is interesting to mention that we were initially unable to detect the corresponding DNA–B cognates of the original SiMoV isolates allocated to subgroup #3 even after exhaustive searches in GenBank. Nevertheless, it was possible to identify a single sequence (read coverage of 353,487) in our searches for a putative cognate DNA–B of SiMoV. This contig displayed an identity of 93.5% with the common region of SiMoV DNA–A, displaying it as iteron of GGAGT (Supplementary Figure S1). According to the literature, for a DNA–B component to be cognate to a DNA–A, an identity equal to or greater than 90% is required [13]. Therefore, this contig is meeting the criteria to be considered a cognate SiMoV DNA–B. This is the first *bona-fide* report of a SiMoV DNA–B, confirming the bipartite nature of this begomovirus. The DNA–B sequence was deposited under the accession number PQ240617, being detected in only one sample (ES–076), which was collected in the Espírito Santo–ES state, Southeast Brazil.

Regarding the helix 4 motif [32], SiMMV isolates presented the following amino acid sequence: SKE I–AL QII REK LPE KFL FQF HNL NSN LDR IFA KAP EPW APP FRL SSF NVP DEM QEW A. SiMoV isolates displayed the following sequence: DKH TAL QII REK LPE KYL FQF HNL NSN LDR IFL KAP EP–W TPP FSL SSF TNV PVE MQE WA. Isolates with the code FN557552 and AJ557450 formerly classified as SiMMV displayed the helix 4 motif similar to that of SiMoV, corroborating the result of the phylogeny and SDT, that these sequences are more likely from SiMoV isolates [27,28]. Therefore, according to our study, 45 SiMMV and 8 SiMoV isolates with complete DNA–A genomes are currently available in the GenBank database, including 2 SiMoV isolates misclassified as SiMMV (Table 2).

Another analysis with the near-palindromic DNA–A segment [ACTT–(N7)–AAGT], which is a structural element conserved in the promoters of the CP gene of *Geminiviridae* members, indicated the clear discrimination of SiMMV and SiMoV isolates. The SiMMV sequences showed the sequence ACTT–GGGCCCT–AAGT, and SiMoV displayed the sequence ACTT–GGTCCCT–AAGT, presenting a difference of one nucleotide at position 7 [33]. Interestingly, an analysis of the presence of “novel” ORFs in DNA–A indicated that both SiMMV and SiMoV have AC5 and AC7, but not V3 and AC6.

The analyses of our SiMoV isolate (PQ240619) with the RDP 5 program showed evidence of recombination events using five statistical methods: RDP statistical (*p*-value = 2.248 × 10^−6^), GENECONV (*p*-value = 5.492 *×* 10^−8^), MaxChi (*p*-value = 2.538 *×* 10^−2^), SiScan (*p*-value = 1.605 × 10^−5^), and 3 Seq (*p*-value = 2.048 *×* 10^−4^). These analyses showed that SiMoV closely resembles (91.5%) the major parent ToYSV and the minor parent SiMMV (99.6%). The initial breakpoint is at nucleotide #647, and the final breakpoint is at nucleotide #901, involving the partial sequence of the coat protein and covering the entire ORF AC5. No strong evidence of recombination was found in the SiMMV genome based upon analyses using the RDP5 program [34].

## 4. Discussion

Malvaceae weeds are widely distributed around the world due to their outstanding seed-dispersal mechanisms [41] and flexible environmental adaptability [42]. Species of the genus *Sida* L. are present in both hemispheres across all continents [43,44]. *Sida* species may also serve as reservoirs of a wide array of begomoviruses [44] and play relevant epidemiological roles since the survival of crop-infecting begomoviruses may occur in these weed hosts [43,44,45,46]. In fact, several begomoviruses are propagated and perpetuated almost indefinitely in these Malvaceae species [43].

SiMoV and SiMMV are major Brazilian begomoviruses displaying a wide geographic distribution [16,17,18,19,20,21,22]. Herein, we provided strong evidence of nomenclatural inaccuracies involving the initial descriptions of isolates from the SiMoV and SiMMV complexes. We showed that *bona fide* SiMMV isolates encompass a complex of strains with identities ranging from 88.8–100%. Even though SiMoV was first deposited in GenBank in 2002, a subgroup of isolates of the SiMMV complex was also misnamed as SiMoV. For example, we also found that partial sequences of two putative soybean-infecting SiMoV are more likely isolates of SiMMV. The isolate AY436328 displayed 96.23% and AY444554 displayed 98.27% identity to SiMMV isolates.

The nomenclatural problems originated with the description of the first SiMMV isolates [14,16]. The accession AJ557450 (NC_077711), which is, in fact, the complete DNA–A component of SiMoV but it was used as the reference isolate of SiMMV. A subsequent publication entitled “In planta cloning of geminiviral DNA: The true Sida micrantha mosaic virus” [16] carried out the characterization of a novel SiMMV isolate (FN557522 = SP77) from an herbarium sample of the Instituto Agronômico de Campinas—IAC (São Paulo-SP, Brazil). Somewhat surprising, our MUSCLE alignments and SDT analyses of NC_077711 and FN557522 isolates indicated that both have erroneous designations in GenBank since they are strains of the reference isolate of SiMoV (NC_004637).

After the initial reports of SiMoV and SiMMV [14,16], the accessions AY090555 (NC_004637) and AJ557450 (NC_077711) were established as the reference DNA–A genomes of each viral species [13]. On the other hand, the cognate DNA–B of SiMoV was assigned to AJ557454 [13]. However, we found that AJ557454 corresponds, in fact, to a cognate DNA–B of SiMMV. For a DNA–B to be cognate to a DNA–A, an identity greater than 90% is required [13]. Herein, we were able to detect for the first time the putative DNA–B component of SiMoV, confirming its bipartite nature. The iterons found between DNA–A and DNA–B components in our isolate ES–076 were GGAGT with IRD: MPSKPRRFRVQ (PQ240617). In addition, our putative DNA–B of SiMoV displayed an identity of 93.5% with the cognate DNA–A (Supplementary Figure S1). Hence, it is the first complete genomic characterization of a SiMoV isolate.

In Brazil, many weed and crop species were identified as reservoirs of Malvaceae-associated begomovirus, mainly SiMMV [20,21]. Species of *Sida* may act as reservoirs of SiMMV and tomato mild mosaic virus (ToMlMV), which were found to be infecting tomato in Central Brazil [40] and snap beans (*Phaseolus vulgaris*) in the state of Goiás and in the Federal District [18]. SiMMV infection of passion fruit (*P. edulis*) was reported in São Fidelis (Rio de Janeiro state), Paragominas (Pará state), Araguari and Patos de Minas (both in Minas Gerais state) [19]. A divergent isolate (with 90% identity to SiMoV) was also reported infecting passion fruit [19]. SiMMV was detected in *Oxalis* species in three tomato production areas: Gama and Rajadinha (Federal District) and Formosa (Goiás State) [20]. Isolates of SiMMV infecting three cotton species (*Gossypium* spp.) were also reported in the state of Goiás [22].

## 5. Conclusions

Isolates of the SiMMV complex play an important epidemiological role under Brazilian conditions, and therefore, the taxonomic position and nomenclature of this begomovirus deserve special attention. SiMoV apparently displays a more restrict host range, being able to infect only *Sida* species thus far. Herein, we provided strong evidence of nomenclatural inaccuracies involving the initial descriptions of isolates from the SiMoV and SiMMV complexes. We observed that the GenBank accessions AJ557450 (NC_077711) and FN557522 (=isolate SP77) described as SiMMV [14,16] are, in fact, isolates of SiMoV according to the current taxonomic criteria.

Herein, the putative DNA–B component of SiMoV was detected for the first time, allowing for the characterization of the complete bipartite genome of this virus. The iterons found between DNA–A and DNA–B were GGAGT with IRD: MPSKPRRFRVQ (PQ240617).

Analyses of other conserved motifs in Rep and CP gene promoters showed consistent differences between SiMMV and SiMoV isolates. We also detected strong evidence of the recombinant nature of SiMoV, involving genomic segments of ToYSV and SiMMV.

In summary, SiMMV-related begomoviruses comprise a complex of strains with a large-spectrum host range, being able to infect members of at least five botanical families (Malvaceae, Solanaceae, Fabaceae, Oxalidaceae, and Passifloraceae), whereas the SiMoV group now comprises eight isolates with host range restricted to Malvaceae species. Reassessments of the taxonomic status of begomoviruses in Neotropical areas are necessary due to the large number of species that have been simultaneously described by distinct research groups, especially after the *B. tabaci* MEAM 1 invasion in the early 1990s [45]. A similar misidentification problem of begomoviruses was reported with two South American begomoviruses (tomato golden vein virus—TGVV and tomato yellow veins streak virus –ToYVSV) in which a reappraisal of the classification status based upon the genome-wide pairwise identity of multiple isolates was able to identify a large number of misnamed isolates in GenBank [31]. The correct identification of isolates of SiMMV and SiMoV is a crucial element for the implementation of effective classical and biotech large-spectrum resistance breeding, for which genetic sources are already available in some SiMMV hosts, such as tomatoes [47,48,49].

## Figures and Tables

**Table 1:** List of 79 foliar samples (isolates) of Malvaceae weeds exhibiting begomovirus–like symptoms in 11 states of Brazil that were analyzed via high-throughput sequencing (HTS). Information is provided about the geographical region/state where the isolate was collected, year of collection, and the respective isolate code.

Macrogeographic Region (Number of Samples)	Brazilian State	Year of Collection	Isolate Code
North (*n* = 21)	Amazonas–AM	2016	AM–028, AM–030, and AM–044.
	Tocantins–TO	2005	TO–016, and TO–025.
		2008	TO–100, TO–105, TO–124, TO–134, TO–175, and TO–229.
		2009	TO–242, TO–250, TO–259, TO–270, TO–273, TO–275, and TO–285.
		2010	TO–304, and TO–307.
		2013	TO–324.
Northest (*n* = 22)	Bahia–BA	2007	BA–004 and BA–018.
		2011	BA–082, BA–088, BA–098, BA–103, BA–107, BA–109, and BA–138.
		2012	BA–169, and BA–170.
		2016	BA–180.
		2019	BA–189, BA–190, BA–192, and BA–193.
	Ceará–CE	2012	CE–060, and CE–061.
		2017	CE–075.
	Pernambuco–PE	2007	PE–006, and PE–009.
		2016	PE–146.
Midwest (*n* = 21)	Distrito Federal–DF	2003	DF–036, and DF–069.
		2009	DF–275.
		2010	DF–358.
		2011	DF–332, and DF–394.
		2019	DF–707, and DF–708.
	Goiás–GO	2003	GO–235, and GO–243.
		2007	GO–366.
		2008	GO–413.
		2009	GO–431, and GO–440.
		2010	GO–460, GO–462, and GO–472.
		2013	GO–548.
		2019	GO–623.
		2020	GO–268, and GO–628.
Southest (*n* = 14)	Espírito Santo–ES	2001	ES–002.
		2011	ES–047.
		2012	ES–072, ES–076, and ES–077.
	Minas Gerais–MG	2002	MG–032.
		2008	MG–059.
	Rio de Janeiro–RJ	2006	RJ–010, RJ–011, RJ–012, RJ–013, and RJ–014.
		2016	RJ–055, and RJ–056.
South (*n* = 1)	Santa Catarina–SC	2010	SC–035.

**Table 2:** The 53 isolates designated as either Sida micrantha mosaic virus (SiMMV = *Begomovirus sidamicranthae*) or Sida mottle virus (SiMoV = *Begomovirus sidavariati*) with complete DNA–A genomes currently available in GenBank. In bold are all eight currently available SiMoV isolates. The two SiMoV isolates (which were found herein to be misclassified as SiMMV) are highlighted in gray.

GeneBank Accession #	Viral Species Associated with the GenBank Accession	Confirmation of the Viral Species	Viral Species After the Present Work
**AJ557450 = NC_077711**	**SiMMV**	**No**	**SiMoV**
AJ557451 = NC_005330	SiMMV	Yes	SiMMV
**AY090555 = NC_004637**	**SiMoV**	**Yes**	**SiMoV**
EU908733	SiMMV	Yes	SiMMV
FJ686693	SiMMV	Yes	SiMMV
FN436003	SiMMV	Yes	SiMMV
FN436005	SiMMV	Yes	SiMMV
**FN557522 (=SP77)**	**SiMMV**	**No**	**SiMoV**
HM357459	SiMMV	Yes	SiMMV
HM585431	SiMMV	Yes	SiMMV
HM585433	SiMMV	Yes	SiMMV
HM585437	SiMMV	Yes	SiMMV
HM585439	SiMMV	Yes	SiMMV
JX415187	SiMMV	Yes	SiMMV
JX415194	SiMMV	Yes	SiMMV
JX415195	SiMMV	Yes	SiMMV
**JX871377**	**SiMoV**	**Yes**	**SiMoV**
**JX871378**	**SiMoV**	**Yes**	**SiMoV**
KC706535	SiMMV	Yes	SiMMV
KC706536	SiMMV	Yes	SiMMV
KC706537	SiMMV	Yes	SiMMV
KU852503	SiMMV	Yes	SiMMV
KX348155	SiMMV	Yes	SiMMV
KX348156	SiMMV	Yes	SiMMV
KX348157	SiMMV	Yes	SiMMV
KX348158	SiMMV	Yes	SiMMV
KX348159	SiMMV	Yes	SiMMV
KX348160	SiMMV	Yes	SiMMV
KX348161	SiMMV	Yes	SiMMV
KX348162	SiMMV	Yes	SiMMV
KX348163	SiMMV	Yes	SiMMV
KX348164	SiMMV	Yes	SiMMV
KX691401	SiMMV	Yes	SiMMV
KX691410	SiMMV	Yes	SiMMV
KY650717	SiMMV	Yes	SiMMV
KY650722	SiMMV	Yes	SiMMV
MF957204	SiMMV	Yes	SiMMV
MT103974	SiMMV	Yes	SiMMV
MT103979	SiMMV	Yes	SiMMV
MT103980	SiMMV	Yes	SiMMV
MT103981	SiMMV	Yes	SiMMV
MT103982	SiMMV	Yes	SiMMV
MT103983	SiMMV	Yes	SiMMV
MT103984	SiMMV	Yes	SiMMV
MT103985	SiMMV	Yes	SiMMV
MT103986	SiMMV	Yes	SiMMV
MT214092	SiMMV	Yes	SiMMV
MT733803	SiMMV	Yes	SiMMV
MT733814	SiMMV	Yes	SiMMV
PQ240611	SiMMV	Yes	SiMMV
**PQ240616**	**SiMoV**	**Yes**	**SiMoV**
**PQ240618**	**SiMoV**	**Yes**	**SiMoV**
**PQ240619**	**SiMoV**	**Yes**	**SiMoV**

## Data Availability

All data generated or analyzed during this study are included in this published article, the Supplementary Materials, and GenBank (accessions: PQ240611, PQ240612, PQ240614, PQ240616, PQ240617, PQ240618, and PQ240619).

## Supplementary Materials

The following supporting information can be downloaded at: https://www.mdpi.com/article/10.3390/v16111796/s1, Supplementary Table S1: PCR primers specific for Sida micrantha mosaic virus (SiMMV = *Begomovirus sidamicranthae*) and primers specific for Sida mottle virus (SiMoV = *Begomovirus sidavariati*). These primers were employed to detect the presence of both viruses in 79 samples of Malvaceae weeds. Supplementary Table S2: Malvaceae samples with the confirmed presence of Sida micrantha mosaic virus (SiMMV = *Begomovirus sidamicranthae*) infection. Supplementary Figure S1: The putative bipartite DNA–A and DNA–B common (intergenic) region (CR) of Sida mottle virus (ES–076 isolate). The CRs of three DNA–A genomes (GenBank accessions PQ240616, PQ240618, and PQ240044) and DNA–B (GenBank accession: PQ240617) displaying the GGAGT core, TATA region, nonanucleotide, and Rep Iteron-Related domain in the motif I (MPSKPRRFRVQ).
