# Supplementary material for: Reexamination of the Sida Micrantha Mosaic Virus and Sida Mottle Virus Complexes: Classification Status, Diversity, Cognate DNA–B Components, and Host Spectrum
Source: Viruses. 2024 Nov 19;16(11):1796. doi: 10.3390/v16111796 (PMC11599112; doi:10.3390/v16111796)
Supplement: Supplementary file 1 [file viruses-16-01796-s001.zip › Table S1.pdf]

Supplementary Table S1. Information about the 53 isolates of Sida micrantha mosaic virus (SiMMV = *Begomovirus sidamicranthae*) and Sida mottle virus (SiMoV = *Begomovirus sidavariati*) available with complete DNA–A genomes in the GenBank database that were employed in the phylogenetic analysis. The tomato leaf curl virus (ToLCV – FM210277) isolate was used as outgroup in this analysis. In **bold** are the isolates characterized in the present work.

Host (botanic family)	GenBank Accession Number(s)	Virus species / DNA–A length (nucleotides)	Geographical origin of the isolate
Glycine max (Fabaceae)	FJ686693	SiMMV / 2669	BR
Glycine max (Fabaceae)	KU852503	SiMMV / 2677	BR
Phaseolus vulgaris (Fabaceae)	HM357459	SiMMV / 2692	Federal District–DF
Abelmoschus esculentus (Malvaceae)	EU908733	SiMMV / 2684	BR
Abelmoschus esculentus (Malvaceae)	FM210277 (outgroup)	ToLCV / 2813	Cameroon
Sida micrantha (Malvaceae)	FN436005	SiMMV / 2691	Mato Grosso do Sul– MS
Sida micrantha (Malvaceae)	FN557522	SiMMV / 2665	São Paulo–SP
Sidastrum micranthum (Malvaceae)	HM585433	SiMMV / 2674	Bolivia
Sidastrum rhombifolia (Malvaceae)	HM585431	SiMMV / 2677	Bolivia
Sidastrum rhombifolia (Malvaceae)	HM585437	SiMMV / 2676	Bolivia
Sidastrum rhombifolia (Malvaceae)	HM585439	SiMMV / 2676	Bolivia
Sidastrum rhombifolia (Malvaceae)	FN436003	SiMMV / 2677	Mato Grosso do Sul– MS
Sida santaremensis (Malvaceae)	JX871377	SiMoV / 2662	BR
Sida santaremensis (Malvaceae)	JX871378	SiMoV / 2662	BR
Sida santaremensis (Malvaceae)	JX415187	SiMMV / 2691	Goiás-GO
Sida santaremensis (Malvaceae)	JX415194	SiMMV / 2691	Goiás-GO
Sida santaremensis (Malvaceae)	JX415195	SiMMV / 2691	Goiás-GO
Sida species (Malvaceae)	AY090555 = NC_004637	SiMoV / 2668	BR
Sida species (Malvaceae)	AJ557450 = NC_077711	SiMMV / 2659	São Paulo–SP
Sida species (Malvaceae)	AJ557451 = NC_005330	SiMMV / 2675	São Paulo–SP
Sida species (Malvaceae)	KX348155	SiMMV / 2678	BR

Sida species (Malvaceae)	KX348157	SiMMV / 2677	BR
Sida species (Malvaceae)	KX348160	SiMMV / 2679	BR
Sida species (Malvaceae)	KX348161	SiMMV / 2677	BR
Sida species (Malvaceae)	KX348162	SiMMV / 2677	BR
Sida species (Malvaceae)	KX348163	SiMMV / 2675	BR
Sida species (Malvaceae)	KX348164	SiMMV / 2678	BR
Sida species (Malvaceae)	KX348156	SiMMV / 2675	BR
Sida species (Malvaceae)	KX348158	SiMMV / 2675	BR
Sida species (Malvaceae)	KX348159	SiMMV / 2675	BR
Sida species (Malvaceae)	**PQ240611**	SiMMV / 2610	BR
Sida species (Malvaceae)	**PQ240616**	SiMoV / 2640	BR
Sida species (Malvaceae)	**PQ240618**	SiMoV / 2688	BR
Sida species (Malvaceae)	**PQ240619**	SiMoV / 2688	BR
Sida spinosa (Malvaceae)	KX691401	SiMMV / 2678	BR
Sida spinosa (Malvaceae)	KX691410	SiMMV / 2678	BR
Oxalis species (Oxalidaceae)	KY650717	SiMMV / 2678	Federal District–DF
Oxalis species (Oxalidaceae)	KY650722	SiMMV / 2685	Federal District–DF
Passiflora species (Passifloraceae)	MF957204	SiMMV / 2677	Minas Gerais–MG
Passiflora edulis (Passifloraceae)	MT103974	SiMMV / 2677	Bahia–BA
Passiflora edulis (Passifloraceae)	MT103979	SiMMV / 2677	Bahia–BA
Passiflora edulis (Passifloraceae)	MT103980	SiMMV / 2677	Bahia–BA
Passiflora edulis (Passifloraceae)	MT103981	SiMMV / 2677	Bahia–BA
Passiflora edulis (Passifloraceae)	MT103982	SiMMV / 2677	Bahia–BA
Passiflora edulis (Passifloraceae)	MT103983	SiMMV / 2678	Bahia–BA
Passiflora edulis (Passifloraceae)	MT103984	SiMMV / 2679	Bahia–BA

Passiflora edulis (Passifloraceae)	MT103985	SiMMV / 2677	Bahia–BA
Passiflora edulis (Passifloraceae)	MT103986	SiMMV / 2677	Bahia–BA
Solanum lycopersicum (Solanaceae)	KC706535	SiMMV / 2675	BR
Solanum lycopersicum (Solanaceae)	KC706536	SiMMV / 2675	BR
Solanum lycopersicum (Solanaceae)	KC706537	SiMMV / 2675	BR
Solanum lycopersicum (Solanaceae)	MT214092	SiMMV / 2679	Federal District–DF
Solanum lycopersicum (Solanaceae)	MT733803	SiMMV / 2676	Federal District–DF
Solanum lycopersicum (Solanaceae)	MT733814	SiMMV / 2691	Federal District–DF

BR = unspecified location in Brazil.
