# Supplementary material for: Reexamination of the Sida Micrantha Mosaic Virus and Sida Mottle Virus Complexes: Classification Status, Diversity, Cognate DNA–B Components, and Host Spectrum
Source: Viruses. 2024 Nov 19;16(11):1796. doi: 10.3390/v16111796 (PMC11599112; doi:10.3390/v16111796)
Supplement: Supplementary file 1 [file viruses-16-01796-s001.zip › Table S2.pdf]

Supplementary Table S2. PCR primers specific for Sida micrantha mosaic virus (SiMMV = *Begomovirus sidamicranthae*) and primers specific for Sida mottle virus (SiMoV = *Begomovirus sidavariati*).These primers were employed to detect the presence of both viruses in 79 samples of Malvaceae weeds.

Viral Species	Primer	Sequence 5′-3′	Annealing Temperature (°C)
Sida micrantha mosaic virus DNA–A	SiMMV_BamHIF	GGATCCCTCATGGCGCCAGATG	58
	SiMMV_BamHIR	CGAAATGCCCAAGCGGGATCC	
Sida mottle virus DNA–A	F3SiMoV	GGAGGGTAATTGAATAGCTTGC	55
	R2SiMoV	CCCTCCAATAAGAAATTCATCAAG	
