# Supplementary material for: Reexamination of the Sida Micrantha Mosaic Virus and Sida Mottle Virus Complexes: Classification Status, Diversity, Cognate DNA–B Components, and Host Spectrum
Source: Viruses. 2024 Nov 19;16(11):1796. doi: 10.3390/v16111796 (PMC11599112; doi:10.3390/v16111796)
Supplement: Supplementary file 1 [file viruses-16-01796-s001.zip › Table S3.pdf]

Supplementary Table S3. Malvaceae samples with the confirmed presence of Sida micrantha mosaic virus (SiMMV = *Begomovirus sidamicranthae*) infection via partial sequencing of SiMMV-specific amplicons.

Positive samples for SiMMV by geographic regions and Brazilian states[1]				
**North**	**Northeast**	**South**	**Southeast**	**Midwest (Central Brazil)**
TO–016, TO–100, TO–105, TO–124, TO–175, TO–229, TO–250, TO–259, TO–275, TO–285, TO–304, and TO–324	BA–082, BA–088, BA–098, BA–169, BA–190, BA–193, and CE–061	SC–035	ES–002, ES–076, MG–032, RJ–010, RJ–011, RJ–012, RJ–013, and RJ–056	DF–069, DF–332, DF–394, DF–707, GO–235, GO–243, GO–440, GO–462, GO–472, GO–548, GO–623, and GO–628

[1]States of Brazil: BA: Bahia; CE: Ceará; DF: Distrito Federal; ES: Espírito Santo; GO: Goiás; MG: Minas Gerais; RJ: Rio de Janeiro; SC: Santa Catarina, and TO: Tocantins.
