# Supplementary figures and images for: Reexamination of the Sida Micrantha Mosaic Virus and Sida Mottle Virus Complexes: Classification Status, Diversity, Cognate DNA–B Components, and Host Spectrum
Source: Viruses. 2024 Nov 19;16(11):1796. doi: 10.3390/v16111796 (PMC11599112; doi:10.3390/v16111796)

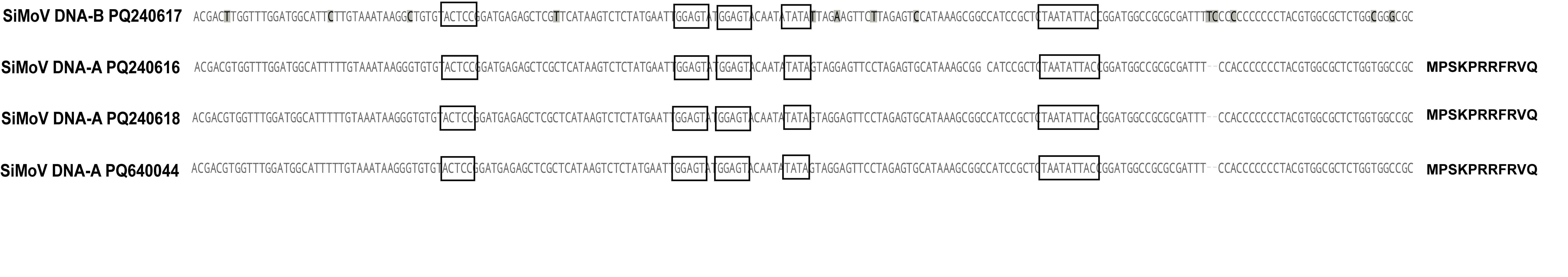

Supplement: Supplementary file 1 [file viruses-16-01796-s001.zip › Figure S1.tif]
